# Dimethylsulfoxide (DMSO) clusters dataset: DFT relative energies, non-covalent interactions, and cartesian coordinates

**DOI:** 10.1016/j.dib.2022.108024

**Published:** 2022-03-07

**Authors:** Alhadji Malloum, Jeanet Conradie

**Affiliations:** a Department of Chemistry, University of the Free State, PO BOX 339, Bloemfontein 9300, South Africa; b Department of Physics, Faculty of Science, University of Maroua, PO BOX 46, Maroua, Cameroon; c Department of Chemistry, UiT - The Arctic University of Norway, Tromsø N-9037, Norway

**Keywords:** Dimethylsulfoxide clusters, Non-covalent interactions, QTAIM analysis, Relative energies

## Abstract

Theoretical understanding of dimethylsulfoxide (DMSO) liquid depends on the understanding of the DMSO clusters. In this work, we provide the structures and the energetics of the DMSO clusters. The structures have been generated using ABCluster and further optimized at the MP2/aug-cc-pVDZ level of theory. The final structures have been optimized at two different levels of theory: PW6B95D3/aug-cc-pVDZ and ωB97XD/aug-cc-pVDZ. The Cartesian coordinates of the structures optimized at the MP2/aug-cc-pVDZ level of theory are also reported. The relative energies of the structures can be used to locate the most favorable structures of the DMSO clusters. The Cartesian coordinates of the structures can be used for further investigations on DMSO clusters. In addition, we report the data related to the quantum theory of atoms in molecule (QTAIM) analysis of the investigated clusters. The QTAIM data reported in this work can be used to understand and determine the nature of non-covalent interactions in DMSO clusters. For further reading and discussion on the data reported here, please report to the original manuscript Malloum and Conradie (2022) [1].

## Specifications Table

**Table:** 

Subject	Chemistry
Specific subject area	Physical and Theoretical Chemistry
Type of data	Figure Table
How data were acquired	Electronic energies and the Cartesian coordinates of the structures are generated using Gaussian 16, at the three different levels of theory. The data related to the quantum theory of atoms in molecule (QTAIM) analysis are generated using the AIMAll program.
Data format	Analyzed Raw
Description of data collection	Raw data are retrieve from the output of the Gaussian calculations. QTAIM data (analyzed data) are from the AIMAll program. Relative energies (analyzed data) are calculated using the electronic energies from Gaussian output files. Geometries are optimized using the resources of the South African Center of High Performance Computing (CHPC). We used our Laboratory clusters to perform QTAIM analysis.
Data source location	Institution: Physical Chemistry Laboratory of the Department of Chemistry, University of the Free State
	City/Town/Region: Bloemfontein
	Country: South Africa
Data accessibility	Repository name: Mendeley Data
	Data identification number (DOI number): 10.17632/bwfjhvkhcz.1
	Direct link to the dataset: https://doi.org/10.17632/bwfjhvkhcz.1
Related research article	A. Malloum, J. Conradie, Non-Covalent Interactions in Dimethylsulfoxide (DMSO) Clusters and DFT Benchmarking, J. Mol. Liq. 350 (2022) 118522. https://doi.org/10.1016/j.molliq.2022.118522.

## Value of the Data

• The data reported in this work are important to understand the hydrogen bond network of the dimethylsulfoxide (DMSO) clusters. This understanding is important for proper theoretical description of the liquid DMSO.
• The relative energies at different levels of theory provide the quantitative data necessary to select relevant structures of the DMSO clusters.
• The QTAIM data can be used to determine covalent and non-covalent bonds of the DMSO clusters, as well as their strength.
• The Cartesian coordinates of the located structures will be of extreme help for further investigations involving DMSO clusters.

## 1 Data Description

**Fig. 1:**
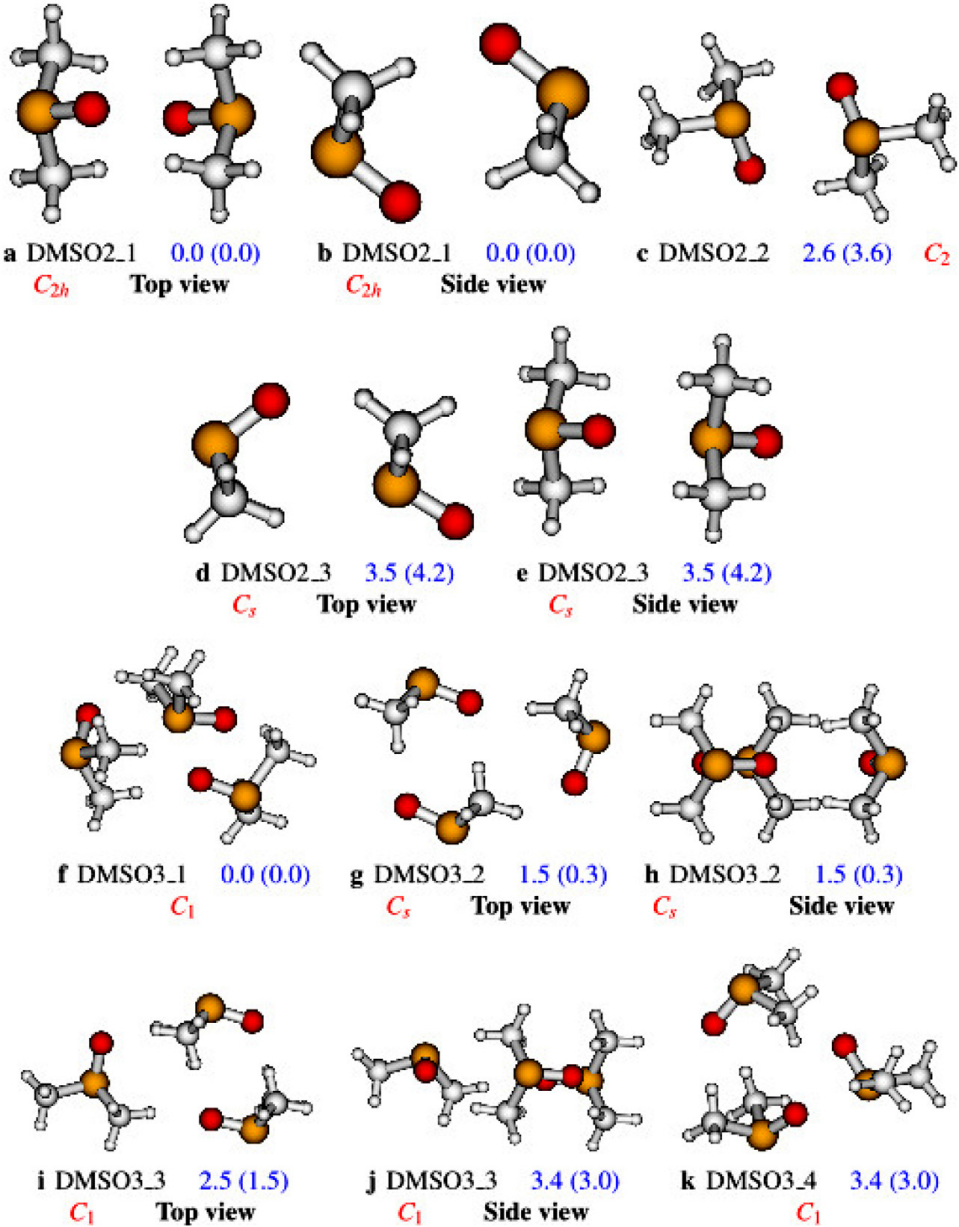
Structures of the DMSO dimer and trimer as optimized at the PW6B95D3/aug-cc-pVDZ level of theory. Numbers represent the zero point corrected relative energies at the PW6B95D3/aug-cc-pVDZ level of theory (and ωB97XD/aug-cc-pVDZ level of theory in brackets).

**Fig. 2:**
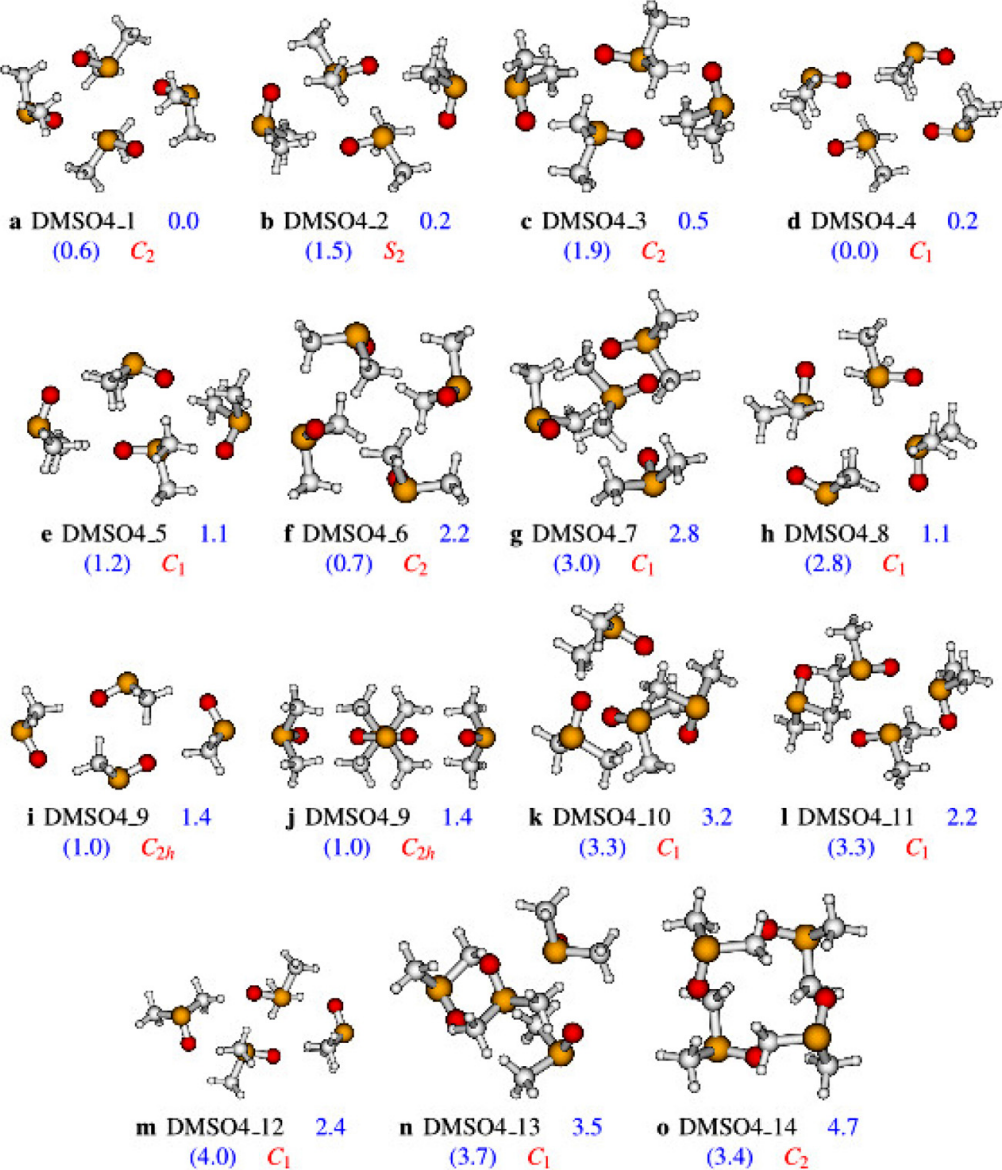
Structures of the DMSO tetramer as optimized at the PW6B95D3/aug-cc-pVDZ level of theory. Numbers represent the zero point corrected relative energies at the PW6B95D3/aug-cc-pVDZ level of theory (and ωB97XD/aug-cc-pVDZ level of theory in brackets).

The data reported in this work are constituted of analyzed data and raw data. The analyzed data are constituted of the structures of the DMSO clusters from n=2 to n=4 and their relative energies as calculated at two different levels of theory: PW6B95D3/aug-cc-pVDZ and ωB97XD/aug-cc-pVDZ. The structures and their relative energies are reported in Figs. 1 and 2. Each sub-caption of the structures in Figs. 1 and 2 reports the name of the structure (in accordance with the naming in the main paper [1]); the relative energies at the PW6B95D3/aug-cc-pVDZ level of theory (and at the ωB97XD/aug-cc-pVDZ level of the theory in brackets); and the symmetry point group of the structure. In addition to the structures and their relative energies, analyzed data are also constituted of data from quantum theory of atoms in molecule (QTAIM) analysis. Data from QTAIM analysis of the DMSO dimers are provided in the supplementary material. These data are constituted of the bond critical points information of the investigated structures of the DMSO clusters. To avoid cumbersome results, the QTAIM analysis data of the DMSO dimers, trimers, and tetramers are reported in the supplementary material. The reader is referred to the supplementary file for clear assessment of the QTAIM data. Each of the tables in the supplementary material has seven columns which describes respectively, the bond critical point’s name (**name**), the bond critical point’s atoms (**Atoms**), the bond critical point’s electron density (ρ), the bond critical point’s Laplacian of the electron density ($\nabla^{2}\rho$), the bond’s ellipticity (**Ellipticity**), the bond critical point’s kinetic energy density (**K**) and the difference between the geometric bond length and the bond path length (**BPL-GBL_I**). As far as raw data of this work are concerned, they are constituted of the structures’ Cartesian coordinates (as provided in Figs. 1 and 2). The MP2/aug-cc-pVDZ level of theory has been used for the optimization of the Cartesian coordinates. These Cartesian coordinates are reported in the supplementary material.

## 2 Experimental Design, Materials and Methods

The methodology (or methods) used to generate the data presented in this paper has been extensively presented in the main article (related research article) [1]. Interested reader are invited to read the related research article for details on the methodology [1]. Nevertheless, we will provide in the next few sentences the methodological procedure used to acquire the data.

Initially, we use ABCluster code [2], [3] to generate structures. We have already used ABCluster to generate the structures of clusters in our previous works [4], [5], [6], [7]. In addition, we have pointed out clearly the efficiency of the ABCluster as compared to density functional theory (DFT) and MP2 [4]. These works have shown that ABCluster is efficient, easy to use and reliable for the generation of initial structures of molecular clusters. The structures of the DMSO clusters generated by ABCluster are optimized at the MP2/aug-cc-pVDZ level of theory. The output files of the optimization, using MP2/aug-cc-pVDZ, have been used to retrieve the optimized Cartesian coordinates of the DMSO clusters which are reported in this work as supplementary material. The structures obtained at the MP2 are re-optimized at two more levels of theory: PW6B95D3/aug-cc-pVDZ and ωB97XD/aug-cc-pVDZ. The output files of these optimizations have been used to retrieve the relative energies of the DMSO clusters reported in Figs. 1 and 2. Furthermore, the images of the structures provided in Figs. 1 and 2 are also retrieved from the output files of the calculations. It is worth noting that all optimization have been performed using the Gaussian 16 suite of programs.

Regarding the data related to the QTAIM analysis, we used the AIMAll program [8] to generate the data. To generate the data with QTAIM through AIMAll, we use the Gaussian checkpoint files containing the orbitals and the electron density information of the structures. AIMAll generates the data reported in the supplementary file as an excel spreadsheet file. The data contains mainly the critical points of the electron density topology and their related information (properties). These properties can be used to understand and quantify the strength of the bondings of the structures [9], [10]. The properties can also be used to differentiate and classify the bondings (covalent and non-covalent) based of the electron density and the Laplacian of the electron density [11], [12].

## CRediT authorship contribution statement

**Alhadji Malloum:** Conceptualization, Methodology, Validation, Formal analysis, Investigation, Data curation, Writing – original draft, Visualization. **Jeanet Conradie:** Resources, Visualization, Writing – review & editing, Supervision, Funding acquisition, Project administration.

## Declaration of Competing Interest

The authors declare that they have no known competing financial interests or personal relationships which have, or could be perceived to have, influenced the work reported in this article.

## Appendix A Supplementary materials

Supplementary Data S1 Supplementary Raw Research Data. This is open data under the CC BY license http://creativecommons.org/licenses/by/4.0/ Supplementary Data S1

Supplementary Data S2 Supplementary Raw Research Data. This is open data under the CC BY license http://creativecommons.org/licenses/by/4.0/ Supplementary Data S2
