# Supplementary material for: Dimethylsulfoxide (DMSO) clusters dataset: DFT relative energies, non-covalent interactions, and cartesian coordinates
Source: Data Brief. 2022 Mar 7;42:108024. doi: 10.1016/j.dib.2022.108024 (PMC8933536; doi:10.1016/j.dib.2022.108024)
Supplement: Supplementary Data S1 — Supplementary Raw Research Data. This is open data under the CC BY license http://creativecommons.org/licenses/by/4.0/ [file mmc1.pdf]

Cartesian coordinates of : DMSO2_1

Atomic number (AN) and Cartesian coordinates

AN	X	Y	Z
C	-0.588901	1.850912	-1.354418
S	0.588740	2.161573	0.000015
H	-1.343627	2.652945	-1.349855
H	-1.041250	0.860172	-1.187858
H	-0.007627	1.886413	-2.286923
C	-0.588740	1.850657	1.354534
H	-1.343418	2.652738	1.350251
H	-0.007341	1.885921	2.286970
H	-1.041179	0.859984	1.187823
O	1.566798	0.947248	-0.000168
C	0.588901	-1.850912	-1.354418
S	-0.588740	-2.161573	0.000015
H	1.343627	-2.652945	-1.349855
H	1.041250	-0.860172	-1.187858
H	0.007627	-1.886413	-2.286923
C	0.588740	-1.850657	1.354534
H	1.343418	-2.652738	1.350251
H	0.007341	-1.885921	2.286970
H	1.041179	-0.859984	1.187823
O	-1.566798	-0.947248	-0.000168

Cartesian coordinates of : DMSO2_2

Atomic number (AN) and Cartesian coordinates

AN	X	Y	Z
C	-1.934879	1.170447	-1.034285
S	-1.709642	0.059762	0.385961
H	-2.545869	2.031800	-0.721665
H	-2.421290	0.597590	-1.838572
H	-0.922423	1.488782	-1.314551
C	-3.468422	-0.382327	0.579552
H	-4.041607	0.514742	0.861828
H	-3.519959	-1.134376	1.379298
H	-3.825602	-0.809811	-0.369492
O	-1.044385	-1.226643	-0.180477
C	1.934879	-1.170445	-1.034287
S	1.709642	-0.059762	0.385960
H	2.545871	-2.031798	-0.721668
H	2.421288	-0.597586	-1.838573
H	0.922423	-1.488782	-1.314553
C	3.468422	0.382325	0.579553
H	4.041606	-0.514745	0.861830
H	3.519958	1.134373	1.379299
H	3.825603	0.809810	-0.369490

O	1.044386	1.226644	-0.180477

Cartesian coordinates of : DMSO2_3

Atomic number (AN) and Cartesian coordinates

AN	X	Y	Z
C	1.713810	1.356156	-0.837509
S	1.618265	0.000000	0.374765
H	0.826840	1.303201	-1.485682
H	2.655807	1.241128	-1.394646
H	1.730850	2.292343	-0.260901
C	1.713852	-1.356174	-0.837484
H	0.826880	-1.303260	-1.485659
H	1.730921	-2.292349	-0.260858
H	2.655845	-1.241128	-1.394624
O	2.963181	0.000029	1.140797
C	-1.929993	-1.356290	0.858450
S	-2.312223	-0.000007	-0.297807
H	-2.654825	-1.339410	1.686935
H	-0.899001	-1.218053	1.216384
H	-2.025044	-2.291146	0.288576
C	-1.929970	1.356338	0.858370
H	-2.654804	1.339521	1.686854
H	-2.025002	2.291161	0.288439
H	-0.898982	1.218103	1.216315
O	-1.159724	-0.000050	-1.338726

Cartesian coordinates of : DMSO3_1

Atomic number (AN) and Cartesian coordinates

AN	X	Y	Z
C	-0.217908	1.410135	1.570901
S	0.002227	1.571041	-0.227322
H	-1.291020	1.305351	1.779741
H	0.219645	2.311394	2.026751
H	0.332564	0.505939	1.863799
C	-1.025226	3.058161	-0.429399
H	-2.067873	2.781861	-0.217286
H	-0.909608	3.378815	-1.474371
H	-0.644701	3.835479	0.250154
O	1.477446	2.073682	-0.400063
C	-1.739955	-1.471440	-1.437793
S	-2.956359	-1.013363	-0.160841
H	-1.833024	-2.553145	-1.623910
H	-0.733555	-1.208447	-1.078884
H	-2.011818	-0.905128	-2.339838

```
  C     -2.109239     -1.847208      1.220175
  H     -2.193497     -2.934935      1.068992
  H     -2.641857     -1.550882      2.134904
  H     -1.054480     -1.535152      1.238585
  O     -2.778910      0.514102      0.082189
  C      2.705282     -0.659555     -1.550430
  S      2.513481     -1.451163      0.076851
  H      3.775435     -0.656653     -1.810828
  H      2.296698      0.362484     -1.472318
  H      2.145073     -1.278304     -2.265828
  C      3.333597     -0.139674      1.035602
  H      4.400497     -0.127102      0.762820
  H      3.218878     -0.407661      2.095557
  H      2.833137      0.811881      0.790824
  O      0.992790     -1.330603      0.417596
 ---------------------------------------------------

Cartesian coordinates of :   DMSO3_2

 ---------------------------------------------------
 Atomic number (AN) and Cartesian coordinates
  AN         X            Y             Z
 ---------------------------------------------------
  C     -2.538326     -1.201970     -1.353475
  S     -3.375034     -0.312312      0.000003
  H     -2.918540     -2.235505     -1.373793
  H     -1.452229     -1.183598     -1.176354
  H     -2.811554     -0.680743     -2.281889
  C     -2.538317     -1.201985      1.353465
  H     -2.918548     -2.235514      1.373790
  H     -2.811524     -0.680753      2.281883
  H     -1.452221     -1.183631      1.176330
  O     -2.791284      1.128209      0.000010
  C      0.090639      1.616228     -1.357132
  S      0.991034      2.422925     -0.000008
  H     -0.974262      1.883766     -1.280978
  H      0.224886      0.530862     -1.235627
  H      0.539085      1.981277     -2.292007
  C      0.090653      1.616254      1.357141
  H     -0.974257      1.883752      1.280970
  H      0.539082      1.981350      2.292005
  H      0.224941      0.530889      1.235678
  O      2.418744      1.780485     -0.000006
  C      2.778091     -1.132243     -1.355401
  S      1.916241     -1.987146      0.000001
  H      3.825157     -1.473514     -1.363726
  H      2.702603     -0.047034     -1.175205
  H      2.276066     -1.435906     -2.285059
  C      2.778092     -1.132242      1.355400
  H      3.825151     -1.473536      1.363748
  H      2.276048     -1.435886      2.285054
  H      2.702632     -0.047033      1.175196
  O      0.459621     -1.413564      0.000004
 ---------------------------------------------------
```

Cartesian coordinates of : DMSO3_3

```
 ---------------------------------------------------
 Atomic number (AN) and Cartesian coordinates
  AN         X            Y            Z
 ---------------------------------------------------
  C      2.303332      1.504926      0.831086
  S      2.960011      0.353823     -0.412468
  H      2.600289      2.528621      0.554444
  H      2.720948      1.210815      1.806093
  H      1.207223      1.422749      0.810780
  C      4.713744      0.804909     -0.215217
  H      4.862045      1.844823     -0.545002
  H      5.289495      0.115888     -0.848703
  H      4.985528      0.670281      0.842572
  O      2.837467     -1.068066      0.203828
  C     -0.042755     -1.489942     -1.277534
  S     -0.936260     -2.365378      0.042155
  H      1.026051     -1.734806     -1.177860
  H     -0.221591     -0.410993     -1.146975
  H     -0.452332     -1.857147     -2.229356
  C     -0.169694     -1.475029      1.429367
  H      0.917247     -1.648517      1.393843
  H     -0.615225     -1.878399      2.349663
  H     -0.403605     -0.407173      1.298305
  O     -2.414508     -1.856977     -0.027137
  C     -2.960883      0.977050     -1.473050
  S     -2.273662      1.924321     -0.079213
  H     -4.030077      1.225980     -1.561977
  H     -2.805176     -0.094394     -1.265182
  H     -2.420778      1.308857     -2.371237
  C     -3.155050      1.020012      1.231111
  H     -4.227357      1.258543      1.152110
  H     -2.756533      1.389092      2.186881
  H     -2.962555     -0.056065      1.087588
  O     -0.778859      1.482047      0.036040
 ---------------------------------------------------
```

Cartesian coordinates of : DMSO3_4

```
 ---------------------------------------------------
 Atomic number (AN) and Cartesian coordinates
  AN         X            Y            Z
 ---------------------------------------------------
  C      1.139636     -1.457850      1.453156
  S      2.454986     -1.820491      0.250043
  H      0.577875     -2.385065      1.646815
  H      0.497770     -0.670602      1.029564
  H      1.632792     -1.105317      2.370179
  C      1.359884     -2.425342     -1.073071
  H      0.994779     -3.424569     -0.788143
  H      1.983771     -2.493196     -1.975482
  H      0.519012     -1.728149     -1.201464
```

```
  O      2.981476     -0.433050     -0.231316
  C      2.094786      2.661002      0.337829
  S      0.349886      2.433960     -0.124447
  H      2.590239      3.250987     -0.449444
  H      2.546941      1.661646      0.439771
  H      2.099299      3.213837      1.287609
  C      0.662869      1.402948     -1.591958
  H      1.024974      2.063685     -2.395506
  H     -0.297912      0.944667     -1.865172
  H      1.409450      0.638459     -1.324811
  O     -0.243246      1.482336      0.961038
  C     -4.143300     -0.794861     -0.577551
  S     -2.525306     -0.035940     -0.210016
  H     -4.943621     -0.185811     -0.128855
  H     -4.144737     -1.821497     -0.181488
  H     -4.244548     -0.809194     -1.671732
  C     -2.599640     -0.270901      1.589977
  H     -3.438750      0.318733      1.990631
  H     -1.647401      0.107250      1.981513
  H     -2.730212     -1.346299      1.784711
  O     -1.486755     -1.069291     -0.733257
 ---------------------------------------------------


Cartesian coordinates of :   DMSO4_1

 ---------------------------------------------------
 Atomic number (AN) and Cartesian coordinates
  AN          X             Y             Z
 ---------------------------------------------------
  C      0.384126     -1.710351     -1.472925
  S      0.003230     -1.768595      0.303853
  H     -0.552535     -1.544615     -2.023140
  H      0.855509     -2.670073     -1.736020
  H      1.093185     -0.878754     -1.603850
  C     -1.015580     -3.277285      0.246658
  H     -1.888077     -3.074936     -0.390745
  H     -1.315845     -3.503113      1.280232
  H     -0.384161     -4.087918     -0.146910
  O      1.338153     -2.217375      0.987391
  C     -0.384154      1.710279     -1.472901
  S     -0.003210      1.768596      0.303865
  H      0.552488      1.544496     -2.023132
  H     -0.855522      2.670000     -1.736031
  H     -1.093236      0.878692     -1.603765
  C      1.015571      3.277302      0.246586
  H      1.888042      3.074957     -0.390852
  H      1.315879      3.503164      1.280141
  H      0.384117      4.087913     -0.146972
  O     -1.338124      2.217375      0.987421
  C      3.229322      0.323591      1.529007
  S      3.798036      0.437913     -0.196026
  H      4.086181      0.017478      2.149900
```

H	2.410276	-0.413461	1.570845
H	2.897179	1.330113	1.819727
C	4.087611	-1.342429	-0.433382
H	4.953904	-1.632757	0.181759
H	4.318671	-1.479126	-1.499387
H	3.174758	-1.881162	-0.130695
O	2.527241	0.779993	-1.039016
C	-3.229345	-0.323574	1.529006
S	-3.798046	-0.437896	-0.196031
H	-4.086203	-0.017439	2.149890
H	-2.410283	0.413461	1.570846
H	-2.897225	-1.330101	1.819736
C	-4.087582	1.342451	-0.433400
H	-4.953884	1.632796	0.181720
H	-4.318616	1.479146	-1.499412
H	-3.174728	1.881171	-0.130694
O	-2.527249	-0.780007	-1.039004

Cartesian coordinates of : DMSO4_2

Atomic number (AN) and Cartesian coordinates

AN	X	Y	Z
C	-1.131386	1.329196	1.591037
S	-0.909317	1.478408	-0.206741
H	-2.196923	1.464214	1.819158
H	-0.495185	2.098357	2.054660
H	-0.797503	0.315772	1.852872
C	-1.564842	3.167230	-0.366404
H	-2.642005	3.135733	-0.150904
H	-1.382077	3.476348	-1.405227
H	-1.011243	3.817947	0.327136
O	0.644902	1.636031	-0.384610
C	1.564843	-3.167230	0.366403
S	0.909317	-1.478409	0.206738
H	2.642006	-3.135732	0.150906
H	1.011246	-3.817948	-0.327139
H	1.382076	-3.476349	1.405225
C	1.131390	-1.329197	-1.591039
H	2.196927	-1.464215	-1.819160
H	0.797507	-0.315772	-1.852875
H	0.495189	-2.098357	-2.054664
O	-0.644902	-1.636032	0.384605
C	-3.267756	-1.086137	-1.451660
S	-4.340099	-0.360229	-0.170118
H	-3.642202	-2.099117	-1.668928
H	-2.231549	-1.112132	-1.080268
H	-3.372184	-0.444968	-2.338510
C	-3.719948	-1.381933	1.204211
H	-4.048015	-2.419698	1.035164
H	-4.181083	-0.984786	2.119546
H	-2.621335	-1.316896	1.231236

```
  O     -3.802789      1.080125      0.087922
  C      3.719949      1.381933     -1.204209
  S      4.340098      0.360230      0.170122
  H      4.048015      2.419699     -1.035160
  H      2.621336      1.316896     -1.231236
  H      4.181087      0.984787     -2.119543
  C      3.267752      1.086138      1.451662
  H      3.642197      2.099117      1.668930
  H      3.372177      0.444968      2.338512
  H      2.231546      1.112133      1.080267
  O      3.802789     -1.080124     -0.087919
 ---------------------------------------------------

Cartesian coordinates of :   DMSO4_3

 ---------------------------------------------------
 Atomic number (AN) and Cartesian coordinates
  AN         X            Y            Z
 ---------------------------------------------------
  C     -3.853421      1.550627      0.405347
  S     -3.994659      0.389520     -0.991135
  H     -4.053343      2.566518      0.029748
  H     -2.838169      1.479651      0.823653
  H     -4.623778      1.258234      1.132721
  C     -2.499582      0.959577     -1.863856
  H     -2.723598      1.940217     -2.312992
  H     -2.300346      0.217240     -2.649679
  H     -1.674834      1.027883     -1.137722
  O     -3.653282     -1.017727     -0.415241
  C      3.853471     -1.550607      0.405356
  S      3.994664     -0.389502     -0.991133
  H      4.053494     -2.566483      0.029774
  H      2.838200     -1.479713      0.823628
  H      4.623778     -1.258142      1.132754
  C      2.499600     -0.959610     -1.863845
  H      2.723631     -1.940263     -2.312947
  H      2.300358     -0.217300     -2.649693
  H      1.674849     -1.027905     -1.137713
  O      3.653248      1.017740     -0.415251
  C      1.505010      3.155410      0.163410
  S      0.855961      1.470171      0.380234
  H      2.589206      3.085141     -0.001478
  H      1.247025      3.738688      1.060084
  H      1.002301      3.574287     -0.719867
  C      1.681301      1.122785      1.961442
  H      2.765399      1.210514      1.809725
  H      1.398588      0.094975      2.227985
  H      1.292169      1.851039      2.689116
  O     -0.653304      1.672291      0.768370
  C     -1.505031     -3.155407      0.163402
  S     -0.855971     -1.470173      0.380236
  H     -2.589223     -3.085124     -0.001504
  H     -1.247068     -3.738689      1.060079
  H     -1.002313     -3.574288     -0.719868
```

```
  C     -1.681307     -1.122796      1.961450
  H     -2.765405     -1.210544      1.809741
  H     -1.398615     -0.094980      2.227994
  H     -1.292159     -1.851042      2.689123
  O      0.653297     -1.672308      0.768358
 ---------------------------------------------------

Cartesian coordinates of :   DMSO4_4

 ---------------------------------------------------
 Atomic number (AN) and Cartesian coordinates
  AN         X            Y            Z
 ---------------------------------------------------
  C      4.514284      0.069119     -1.023515
  S      3.737558     -1.318584     -0.139583
  H      5.578755      0.107544     -0.743791
  H      3.976369      0.987097     -0.735603
  H      4.414261     -0.148969     -2.096316
  C      3.898004     -0.615040      1.530222
  H      4.967236     -0.570441      1.790283
  H      3.373451     -1.301873      2.209593
  H      3.434158      0.385537      1.509475
  O      2.214003     -1.224061     -0.474876
  C      0.218317      3.380571      0.406690
  S      1.082146      1.783541      0.307142
  H     -0.832216      3.204848      0.133129
  H      0.723461      4.083768     -0.272298
  H      0.308018      3.725188      1.446530
  C      0.875197      1.548111     -1.482402
  H     -0.203076      1.514850     -1.696799
  H      1.365407      0.594083     -1.719591
  H      1.373679      2.392958     -1.982093
  O      2.593948      2.132433      0.511674
  C     -4.257730      0.012199     -1.220148
  S     -3.571540      1.266463     -0.095652
  H     -5.350238     -0.010889     -1.082757
  H     -3.793173     -0.956164     -0.970324
  H     -4.012865      0.342073     -2.239718
  C     -3.915969      0.370798      1.449759
  H     -5.006382      0.362386      1.604493
  H     -3.425701      0.938771      2.253278
  H     -3.507999     -0.648753      1.349922
  O     -2.019627      1.197519     -0.285798
  C     -0.794309     -1.750391     -1.371885
  S     -1.259803     -2.603047      0.164716
  H      0.304614     -1.759907     -1.441012
  H     -1.194295     -0.726472     -1.313396
  H     -1.250611     -2.316200     -2.196438
  C     -0.504355     -1.391952      1.289274
  H      0.578666     -1.383551      1.089054
  H     -0.725770     -1.726214      2.312855
  H     -0.952317     -0.409854      1.075320
  O     -2.808056     -2.415176      0.306783
 ---------------------------------------------------
```

Cartesian coordinates of :   DMSO4_5

```
 ---------------------------------------------------
 Atomic number (AN) and Cartesian coordinates
  AN         X            Y            Z
 ---------------------------------------------------
  C      3.571332      1.167500     -1.583455
  S      4.602526      0.297826     -0.357990
  H      3.927283      2.207477     -1.655121
  H      2.520372      1.129336     -1.255825
  H      3.725433      0.649580     -2.540755
  C      3.940687      1.173288      1.097676
  H      4.305144      2.212274      1.067112
  H      4.346106      0.657797      1.979912
  H      2.840710      1.139341      1.069158
  O      4.054753     -1.156069     -0.281189
  C     -3.204204     -0.720046     -1.732754
  S     -4.372857      0.074257     -0.583524
  H     -3.674501     -1.641920     -2.110054
  H     -2.268734     -0.936810     -1.194309
  H     -3.049830     -0.007931     -2.555825
  C     -4.198873     -1.149870      0.753441
  H     -4.661254     -2.091879      0.418950
  H     -4.747167     -0.745942      1.616222
  H     -3.130324     -1.294628      0.972510
  O     -3.669179      1.368887     -0.074895
  C      1.025534     -1.683390     -1.209075
  S      0.342252     -2.471615      0.276807
  H      2.091181     -1.947017     -1.288766
  H      0.908537     -0.596465     -1.079967
  H      0.443616     -2.062257     -2.061052
  C      1.427026     -1.653548      1.480483
  H      2.469887     -1.918211      1.248535
  H      1.122466     -2.012790      2.473567
  H      1.272431     -0.569490      1.368978
  O     -1.069191     -1.815141      0.474428
  C     -1.266803      1.087454      1.816664
  S     -0.741539      1.367868      0.099705
  H     -2.322805      1.375744      1.901549
  H     -0.605919      1.695883      2.452742
  H     -1.135257      0.013106      2.003297
  C     -1.157756      3.136128      0.030421
  H     -2.251277      3.230868      0.080374
  H     -0.776079      3.509673     -0.930277
  H     -0.648419      3.640727      0.865067
  O      0.832444      1.342954      0.165856
 ---------------------------------------------------
```

Cartesian coordinates of :   DMSO4_6

```
 ---------------------------------------------------
 Atomic number (AN) and Cartesian coordinates
  AN         X            Y            Z
```

```
 ---------------------------------------------------
  C     -2.787273     -2.734443      0.904338
  S     -3.036870     -0.944180      0.685450
  H     -3.086384     -3.001514      1.930114
  H     -1.722038     -2.955689      0.732615
  H     -3.438210     -3.236482      0.174957
  C     -1.676661     -0.416973      1.765410
  H     -1.955764     -0.642776      2.806942
  H     -1.553353      0.666864      1.614660
  H     -0.776769     -0.971120      1.457899
  O     -2.566942     -0.625407     -0.771361
  C      0.416989     -1.676649     -1.765412
  S      0.944176     -3.036867     -0.685451
  H      0.642791     -1.955754     -2.806944
  H      0.971148     -0.776763     -1.457900
  H     -0.666846     -1.553328     -1.614667
  C      2.734442     -2.787288     -0.904326
  H      3.001514     -3.086398     -1.930103
  H      3.236472     -3.438233     -0.174946
  H      2.955700     -1.722056     -0.732601
  O      0.625397     -2.566941      0.771359
  C     -0.416973      1.676653     -1.765403
  S     -0.944184      3.036869     -0.685453
  H     -0.642773      1.955747     -2.806938
  H     -0.971121      0.776761     -1.457890
  H      0.666863      1.553346     -1.614650
  C     -2.734446      2.787269     -0.904343
  H     -3.001512      3.086375     -1.930122
  H     -3.236488      3.438211     -0.174967
  H     -2.955695      1.722036     -0.732617
  O     -0.625410      2.566951      0.771361
  C      2.787288      2.734442      0.904335
  S      3.036871      0.944176      0.685454
  H      3.086386      3.001509      1.930116
  H      1.722058      2.955700      0.732599
  H      3.438241      3.236475      0.174963
  C      1.676646      0.416986      1.765402
  H      1.955738      0.642791      2.806937
  H      1.553331     -0.666850      1.614657
  H      0.776760      0.971140      1.457882
  O      2.566953      0.625405     -0.771360
 ---------------------------------------------------

Cartesian coordinates of :   DMSO4_7

 ---------------------------------------------------
 Atomic number (AN) and Cartesian coordinates
  AN          X             Y             Z
 ---------------------------------------------------
  C     -2.199015     -2.479872      0.601787
  S     -3.061954     -1.522671     -0.686628
  H     -2.866373     -2.576357      1.472770
  H     -1.251803     -1.991602      0.872935
  H     -2.002224     -3.472226      0.171568
```

```
  C     -3.158289      0.047349      0.232769
  H     -3.724733     -0.117698      1.162705
  H     -3.700679      0.756686     -0.408918
  H     -2.142606      0.423544      0.428451
  O     -2.030365     -1.285436     -1.824449
  C     -0.779540      0.470497      2.949946
  S      0.351591     -0.004562      1.600620
  H     -1.145422      1.489021      2.754487
  H     -0.230947      0.393047      3.900323
  H     -1.606055     -0.254284      2.936760
  C      1.674368      1.159689      2.045236
  H      1.257282      2.175671      2.013450
  H      2.464156      1.023329      1.295104
  H      2.018974      0.877523      3.051595
  O      0.886731     -1.415540      2.023514
  C      1.260221      3.318058     -0.882222
  S     -0.535581      3.014045     -0.817383
  H      1.504655      3.701525     -1.885115
  H      1.788461      2.373444     -0.683623
  H      1.479400      4.081948     -0.122727
  C     -0.514214      1.566171     -1.914539
  H     -0.231750      1.917276     -2.919892
  H     -1.518491      1.124731     -1.936519
  H      0.221927      0.848902     -1.521668
  O     -0.826099      2.475989      0.621648
  C      3.497133     -2.029073      0.051584
  S      2.574790     -1.294393     -1.335507
  H      3.669116     -3.094910     -0.165681
  H      2.884226     -1.888471      0.957049
  H      4.455406     -1.495030      0.117806
  C      1.065476     -2.283633     -1.111760
  H      1.274296     -3.300816     -1.480153
  H      0.259765     -1.819328     -1.700952
  H      0.824291     -2.279563     -0.037070
  O      2.217326      0.151215     -0.856601
 ---------------------------------------------------

Cartesian coordinates of :   DMSO4_8

 ---------------------------------------------------
 Atomic number (AN) and Cartesian coordinates
  AN          X             Y             Z
 ---------------------------------------------------
  C      2.705538     -0.579689      1.623010
  S      2.378563     -0.837738     -0.146592
  H      2.992742      0.471167      1.768288
  H      3.497140     -1.285236      1.916353
  H      1.770445     -0.817116      2.151089
  C      4.052021     -0.459497     -0.743981
  H      4.263370      0.597574     -0.533338
  H      4.045694     -0.652587     -1.825964
  H      4.746962     -1.148014     -0.240318
  O      2.223837     -2.383971     -0.308970
  C     -3.259176      0.711382      1.554815
```

```
  S     -2.538509      0.896312     -0.104451
  H     -3.422328     -0.358777      1.740802
  H     -4.185215      1.305344      1.577643
  H     -2.527613      1.124485      2.264158
  C     -3.940649      0.208854     -1.033868
  H     -4.039992     -0.851595     -0.762594
  H     -3.696769      0.325135     -2.099573
  H     -4.832588      0.802458     -0.783877
  O     -2.553524      2.430628     -0.389551
  C     -0.409297     -3.875933      0.419246
  S     -0.826267     -2.117903      0.233277
  H      0.648634     -4.002523      0.151764
  H     -1.090182     -4.450882     -0.226021
  H     -0.587085     -4.125481      1.474797
  C     -0.627507     -2.031904     -1.573303
  H      0.406080     -2.316988     -1.816092
  H     -0.837398     -0.993496     -1.867471
  H     -1.372001     -2.712861     -2.012926
  O     -2.367476     -2.038342      0.477087
  C      0.519628      2.265824     -1.426591
  S      1.389307      2.951131      0.015795
  H     -0.534043      2.590596     -1.385396
  H      0.610852      1.170702     -1.365652
  H      1.032232      2.647663     -2.320304
  C      0.298594      2.214981      1.272621
  H     -0.699723      2.664196      1.153212
  H      0.735936      2.454628      2.252058
  H      0.268032      1.125879      1.110801
  O      2.737462      2.172034      0.116233
 ---------------------------------------------------

Cartesian coordinates of :   DMSO4_9

 ---------------------------------------------------
 Atomic number (AN) and Cartesian coordinates
  AN         X             Y             Z
 ---------------------------------------------------
  C     -1.156580     -1.523477      1.356976
  S     -0.205778     -2.264705     -0.000035
  H     -2.193188     -1.887603      1.288767
  H     -1.123857     -0.431550      1.221612
  H     -0.670820     -1.838757      2.291145
  C     -1.156608     -1.523478     -1.357028
  H     -2.193214     -1.887606     -1.288804
  H     -0.670860     -1.838758     -2.291204
  H     -1.123878     -0.431551     -1.221663
  O      1.176631     -1.516428     -0.000054
  C      4.096239     -0.948509      1.353947
  S      4.814622      0.037704      0.000055
  H      4.606654     -1.924294      1.375999
  H      3.016978     -1.071713      1.174853
  H      4.300530     -0.395311      2.281643
  C      4.096339     -0.948488     -1.353903
  H      4.606705     -1.924299     -1.375896
```

```
  H      4.300752     -0.395308     -2.281583
  H      3.017053     -1.071634     -1.174922
  O      4.055029      1.395612      0.000030
  C      1.156576      1.523466      1.356973
  S      0.205762      2.264684     -0.000036
  H      2.193186      1.887585      1.288749
  H      1.123841      0.431538      1.221614
  H      0.670825      1.838755      2.291144
  C      1.156592      1.523457     -1.357031
  H      2.193187      1.887620     -1.288835
  H      0.670816      1.838705     -2.291204
  H      1.123893      0.431531     -1.221645
  O     -1.176637      1.516391     -0.000042
  C     -4.096226      0.948509      1.353958
  S     -4.814610     -0.037677      0.000048
  H     -4.606608      1.924311      1.376001
  H     -3.016956      1.071677      1.174893
  H     -4.300559      0.395318      2.281649
  C     -4.096311      0.948527     -1.353895
  H     -4.606691      1.924330     -1.375893
  H     -4.300703      0.395345     -2.281579
  H     -3.017029      1.071688     -1.174896
  O     -4.055037     -1.395596      0.000013
 ---------------------------------------------------

Cartesian coordinates of :   DMSO4_10

 ---------------------------------------------------
 Atomic number (AN) and Cartesian coordinates
  AN         X             Y             Z
 ---------------------------------------------------
  C      2.925560      1.707975     -0.211911
  S      2.426331      1.463647      1.522676
  H      3.127098      2.780500     -0.360869
  H      2.150866      1.342189     -0.904818
  H      3.855110      1.136163     -0.347556
  C      0.874251      2.414784      1.456182
  H      1.099021      3.433450      1.103399
  H      0.480201      2.458624      2.481410
  H      0.159304      1.896672      0.799204
  O      2.005370     -0.031436      1.652075
  C      2.088420     -1.780054     -1.128579
  S      0.748333     -2.930652     -0.706909
  H      3.010723     -2.367572     -1.261919
  H      2.177283     -1.059500     -0.301134
  H      1.794874     -1.270416     -2.058737
  C      1.268368     -3.211482      1.016752
  H      2.233652     -3.741399      1.005793
  H      0.497744     -3.837734      1.487886
  H      1.359430     -2.231499      1.510318
  O     -0.544552     -2.058966     -0.608391
  C     -3.367934     -1.106169      0.656968
  S     -2.733818      0.315806      1.603729
  H     -3.693290     -1.873702      1.376745
```

```
  H     -2.569047     -1.487852      0.003489
  H     -4.232161     -0.740436      0.084395
  C     -1.197656     -0.490970      2.143811
  H     -1.468193     -1.368219      2.752957
  H     -0.632051      0.227812      2.749541
  H     -0.636384     -0.783194      1.243962
  O     -2.303492      1.382891      0.547915
  C     -0.878761      2.646154     -1.836820
  S     -0.350557      0.911126     -1.640335
  H     -1.744105      2.828318     -1.183482
  H     -1.105453      2.813853     -2.900382
  H     -0.022544      3.268038     -1.537123
  C     -1.824394      0.170314     -2.401299
  H     -2.702467      0.501270     -1.829034
  H     -1.688177     -0.917220     -2.336498
  H     -1.851633      0.514170     -3.446268
  O      0.796232      0.734449     -2.687659
 ---------------------------------------------------

Cartesian coordinates of :   DMSO4_11

 ---------------------------------------------------
 Atomic number (AN) and Cartesian coordinates
  AN         X             Y             Z
 ---------------------------------------------------
  C      3.915654      1.352779     -0.049696
  S      3.779234      0.159732      1.320082
  H      4.143322      2.342116      0.377644
  H      2.962235      1.372041     -0.599676
  H      4.751657      1.010914     -0.676085
  C      2.209504      0.810037      1.979907
  H      2.415064      1.781236      2.457280
  H      1.859968      0.082440      2.725795
  H      1.501313      0.912671      1.145242
  O      3.436680     -1.210879      0.664186
  C      1.224370     -3.143933     -0.306546
  S      0.744020     -1.414685     -0.604157
  H      2.267395     -3.155057      0.039349
  H      1.079592     -3.704579     -1.242313
  H      0.550161     -3.525492      0.473437
  C      1.849570     -1.122852     -2.016738
  H      2.884069     -1.277856     -1.681380
  H      1.678251     -0.079565     -2.316704
  H      1.548903     -1.823994     -2.809917
  O     -0.686853     -1.500379     -1.238481
  C     -4.317108     -0.938154      2.083411
  S     -3.009820     -0.467448      0.902726
  H     -4.222562     -2.009069      2.321713
  H     -5.293142     -0.707612      1.630995
  H     -4.161936     -0.331334      2.986373
  C     -3.597069     -1.496365     -0.473354
  H     -3.525034     -2.555242     -0.181131
  H     -2.913305     -1.306974     -1.309716
  H     -4.632141     -1.198774     -0.698564
```

```
  O     -3.356007      0.998617      0.502500
  C     -1.221320      3.257974     -0.062228
  S     -0.657701      1.585586     -0.502311
  H     -2.295745      3.204169      0.162534
  H     -0.994173      3.930658     -0.902978
  H     -0.652275      3.558139      0.829067
  C     -1.595460      1.444483     -2.051608
  H     -2.664860      1.530411     -1.814411
  H     -1.354306      0.450656     -2.453133
  H     -1.242156      2.247131     -2.716292
  O      0.832320      1.769917     -0.956637
 ---------------------------------------------------

Cartesian coordinates of :   DMSO4_12

 ---------------------------------------------------
 Atomic number (AN) and Cartesian coordinates
  AN         X            Y            Z
 ---------------------------------------------------
  C     -3.448834      1.161657      1.350624
  S     -4.476914      0.431276      0.035621
  H     -3.785576      2.199440      1.503023
  H     -2.391966      1.122283      1.044981
  H     -3.637305      0.565183      2.254585
  C     -3.732875      1.371194     -1.336055
  H     -4.028552      2.426729     -1.227979
  H     -4.153504      0.954591     -2.262230
  H     -2.638079      1.263737     -1.292911
  O     -3.993076     -1.039846     -0.136678
  C     -1.248334     -1.436370     -1.491070
  S     -1.104873     -1.490530      0.320795
  H     -2.307395     -1.551511     -1.757706
  H     -0.619037     -2.248691     -1.885469
  H     -0.869296     -0.450637     -1.793324
  C     -1.804388     -3.156471      0.535392
  H     -2.869826     -3.114000      0.268522
  H     -1.676466     -3.414996      1.596059
  H     -1.235594     -3.854696     -0.097113
  O      0.433336     -1.664553      0.579158
  C      1.481822      3.093948     -0.386859
  S      0.860334      1.396732     -0.187859
  H      2.562942      3.085262     -0.190375
  H      0.930632      3.744354      0.308912
  H      1.273406      3.384809     -1.426018
  C      1.123062      1.276850      1.606549
  H      2.180596      1.489183      1.814422
  H      0.858986      0.245626      1.879807
  H      0.448254      2.010883      2.073122
  O     -0.697596      1.526346     -0.345830
  C      3.199886     -1.379963     -0.901480
  S      4.099096     -0.419715      0.350001
  H      3.587600     -2.410830     -0.888934
  H      3.370456     -0.892634     -1.873750
  H      2.136397     -1.375160     -0.615034
```

```
  C      5.773058     -0.724774     -0.298820
  H      6.014582     -1.793585     -0.192541
  H      6.461892     -0.114728      0.301916
  H      5.795606     -0.405570     -1.351717
  O      3.818407      1.079843      0.033743
 ---------------------------------------------------

Cartesian coordinates of :   DMSO4_13

 ---------------------------------------------------
 Atomic number (AN) and Cartesian coordinates
  AN          X             Y             Z
 ---------------------------------------------------
  C     -0.938577      0.362253      2.759068
  S      0.401963      0.279975      1.534415
  H     -1.358504      1.378085      2.755502
  H     -0.535061      0.064984      3.738444
  H     -1.710175     -0.341531      2.421944
  C      1.525069      1.435577      2.382668
  H      1.024724      2.410549      2.490692
  H      2.407281      1.527909      1.735143
  H      1.780547      1.004985      3.362440
  O      1.047763     -1.130211      1.755368
  C     -0.351490      3.352122     -0.401200
  S     -1.782923      2.367098     -0.959667
  H     -0.007827      3.973835     -1.242607
  H      0.444506      2.663441     -0.080864
  H     -0.705513      3.988718      0.422284
  C     -0.825790      1.276163     -2.055012
  H     -0.532782      1.867019     -2.937759
  H     -1.475209      0.435799     -2.341283
  H      0.061405      0.927996     -1.505389
  O     -2.215114      1.506060      0.267172
  C     -3.289193     -1.545443      0.306156
  S     -1.576286     -1.654537     -0.299758
  H     -3.308480     -1.727444      1.392258
  H     -3.875409     -2.303232     -0.235205
  H     -3.629188     -0.524825      0.083361
  C     -1.207005     -3.302597      0.378357
  H     -1.155759     -3.229943      1.475404
  H     -0.220935     -3.582937     -0.016868
  H     -1.978489     -4.009590      0.038035
  O     -1.694566     -1.849351     -1.838375
  C      3.898301     -0.924891      0.075158
  S      2.958023     -0.204629     -1.308956
  H      4.357308     -1.867195     -0.262277
  H      3.189264     -1.084801      0.903771
  H      4.678694     -0.197202      0.339270
  C      1.705398     -1.524567     -1.383957
  H      2.205030     -2.446063     -1.722668
  H      0.930716     -1.232547     -2.107019
  H      1.293180     -1.633297     -0.367370
  O      2.237913      1.043387     -0.704566
 ---------------------------------------------------
```

```
Cartesian coordinates of :   DMSO4_14

 ---------------------------------------------------
 Atomic number (AN) and Cartesian coordinates
  AN        X            Y            Z
 ---------------------------------------------------
  C     -2.404803     2.724577    -0.501826
  S     -2.048827     1.606477    -1.897300
  H     -2.265339     3.759718    -0.851020
  H     -1.722980     2.493158     0.330480
  H     -3.457293     2.557379    -0.232210
  C     -0.248177     1.845741    -1.876515
  H     -0.040890     2.906276    -2.091734
  H      0.189810     1.208609    -2.655181
  H      0.116786     1.560201    -0.879165
  O     -2.289965     0.159807    -1.363321
  C      2.404782    -2.724634    -0.501838
  S      2.048765    -1.606530    -1.897297
  H      2.265264    -3.759771    -0.851023
  H      1.723010    -2.493191     0.330503
  H      3.457291    -2.557471    -0.232276
  C      0.248120    -1.845807    -1.876481
  H      0.040839    -2.906333    -2.091752
  H     -0.189885    -1.208643    -2.655110
  H     -0.116840    -1.560314    -0.879115
  O      2.289905    -0.159863    -1.363315
  C      2.099220     0.220687     1.862047
  S      1.621293     1.972817     1.901728
  H      3.126446     0.139188     2.252374
  H      2.029134    -0.122383     0.819112
  H      1.392193    -0.328756     2.497781
  C      2.696262     2.494196     0.525483
  H      3.743953     2.411474     0.854671
  H      2.448834     3.543327     0.310729
  H      2.501205     1.839966    -0.338032
  O      0.164706     2.029177     1.345100
  C     -2.099155    -0.220616     1.862092
  S     -1.621227    -1.972743     1.901823
  H     -3.126367    -0.139104     2.252452
  H     -2.029111     0.122418     0.819143
  H     -1.392110     0.328857     2.497782
  C     -2.696242    -2.494173     0.525635
  H     -3.743920    -2.411447     0.854860
  H     -2.448814    -3.543310     0.310911
  H     -2.501222    -1.839974    -0.337912
  O     -0.164659    -2.029124     1.345148
 ---------------------------------------------------
```
